# Toxicity of Recombinant Necrosis and Ethylene-Inducing Proteins (NLPs) from *Neofusicoccum parvum*

**DOI:** 10.3390/toxins12040235

**Published:** 2020-04-07

**Authors:** Forough Nazar Pour, Rebeca Cobos, Juan José Rubio Coque, João Serôdio, Artur Alves, Carina Félix, Vanessa Ferreira, Ana Cristina Esteves, Ana Sofia Duarte

**Affiliations:** 1 CESAM-Centre for Environmental and Marine Studies, Department of Biology, University of Aveiro, Campus Universitário de Santiago, 3810-193 Aveiro, Portugal; forough.nazarpour84@gmail.com (F.N.P.); jserodio@ua.pt (J.S.); artur.alves@ua.pt (A.A.); carinafelix89@gmail.com (C.F.); fvanessa@ua.pt (V.F.); 2 Instituto de Investigación de la Viña y el Vino (IIVV), Escuela de Ingeniería Agraria, Universidad de León, Avda. Portugal, 41, 24009 León, Spain; rebeca.cobos@unileon.es (R.C.); jjrubc@unileon.es (J.J.R.C.); 3 Faculty of Dental Medicine, Center for Interdisciplinary Research in Health, Universidade Católica Portuguesa, Estrada da Circunvalação, 3504-505 Viseu, Spain; asduarte@viseu.ucp.pt

**Keywords:** Botryosphaeriaceae, Botryosphaeria dieback, necrosis and ethylene-inducing proteins (NLPs), phytotoxicity, cytotoxicity

## Abstract

*Neofusicoccum parvum* is a fungal pathogen associated with a wide range of plant hosts. Despite being widely studied, the molecular mechanism of infection of *N. parvum* is still far from being understood. Analysis of *N. parvum* genome lead to the identification of six putative genes encoding necrosis and ethylene-inducing proteins (NLPs). The sequence of NLPs genes (NprvNep 1-6) were analyzed and four of the six NLP genes were successfully cloned, expressed in *E. coli* and purified by affinity chromatography. Pure recombinant proteins were characterized according to their phytotoxic and cytotoxic effects to tomato leaves and to mammalian Vero cells, respectively. These assays revealed that all NprvNeps tested are cytotoxic to Vero cells and also induce cell death in tomato leaves. NprvNep2 was the most toxic to Vero cells, followed by NprvNep1 and 3. NprvNep4 induced weaker, but, nevertheless, still significant toxic effects to Vero cells. A similar trend of toxicity was observed in tomato leaves: the most toxic was NprvNep 2 and the least toxic NprvNep 4. This study describes for the first time an overview of the NLP gene family of *N. parvum* and provides additional insights into its pathogenicity mechanism.

## 1. Introduction

*Neofusicoccum parvum* is known as a plurivorous fungal pathogen infecting a wide range of plant hosts [1]. This fungus is able to colonize woody tissue through pruning wounds and sites of mechanical or natural injuries [1,2,3], causing internal cankers in the host. The infection also causes characteristic foliar symptoms, although *N. parvum* has not been isolated from the leaves of infected plants [4,5,6]. Several studies have shown that *N. parvum* pathogenicity is multi factorial and involves the production of phytotoxins and extracellular proteins with phytotoxic properties leading to the expression of symptoms in distal tissues [7,8,9]. However, full understanding of the pathogenicity mechanism is still far from being accomplished. Most studies concerning *N. parvum* pathogenicity were conducted on grapevines [4,5,6,7,8,9], but similar symptoms are found in many other species [10,11,12].

Secretome analysis of *Diplodia seriata*, another member of the family Botryosphaeriaceae, revealed the expression of necrosis and ethylene-inducing proteins [13], whose involvement in the infection mechanism was suggested. These proteins are necrotic elicitors connected to a transduction cascade that causes cell death at the site of infection, thereby limiting the spread of the pathogen [14]. Nep1 (necrosis and ethylene-inducing peptide 1) was first isolated from *Fusarium oxysporum* culture filtrates as a 24-kDa protein [15,16]. Over the last decade, several Nep1-like proteins (NLPs) have been found in a diversity of plant-associated microorganisms, such as bacteria, fungi, and oomycetes [17,18]. NLPs cause necrosis and ethylene production only in dicotyledonous plants [19,20,21,22,23,24]. All monocotyledonous plants tested so far are insensitive to NLPs [16,25,26,27,28], suggesting that NLP cytotoxicity requires a dicot-specific target protein or membrane architecture. Recently, glycosylinositol phosphorylceramide (GIPC) sphingolipids were identified as NLP toxin receptors in tobacco [29]. Most monocot GIPCs have three hexose units whereas dicot GIPCs have only two hexose units, thus the difference in the length of GIPC head group may be the cause of insensitivity of monocot plants to NLPs [29]. Likewise, the sensitivity of other organisms like animals, fungi and bacteria to NLPs has not been thoroughly investigated. It is known that the NLP homologue from *Vibrio pommerensis* CH-291 possesses some hemolytic activity against human and animal erythrocytes [30]. On the other hand, Qutob et al. [25] suggested that different cell and tissue types from mammalian and lower plant cell lines, and *Pichia pastoris* spheroplasts are not affected by NLPs.

NLPs are classified into two groups, named type 1 and type 2, according to the presence of either two or four conserved cysteine residues [17]. An additional third type of NLPs—that possess six conserved cysteine residues - has been characterized in Ascomycete fungi [31]. Several studies have identified the key residues of NLPs for their cell-death-inducing activity [20,32]. A conserved 24-aa peptide in type 1 NLPs was identified as being potentially involved in triggering plant immunity responses [33,34] and it includes the conserved regions I and II. Most of the identified NLPs contain a signal peptide, indicating that they are secreted. The expression of NLPs lacking the secretion signal peptide did not induce plant cell death [25,35], consistent with their extracellular activity.

Nonetheless, the exact mechanism by which NLPs cause necrosis is not clear. Induction of necrosis in plants exposed to NLPs can be accompanied by production of ethylene, superoxide anions, overexpression of transcripts coding pathogenesis-related proteins and induction of programmed cell death [20,22,36,37,38]. However, in some plants for example, necrosis induction did not occur by ethylene production [16,36], indicating that other mechanisms may be involved.

Several studies suggested the contribution of NLPs to the virulence of plant pathogens. For example, MpNEP1 and MpNEP2, from *Moniliophthora perniciosa*, were able to induce necrosis and ethylene emission in tobacco and cacao leaves [39]. Furthermore, a drastic increase in virulence of the fungus *Colletotrichum coccodes* towards *Abutilon theophrasti* was observed after overexpression of Nep1 from *Fusarium* sp. [40]. Similarly, individual deletion of two NLP genes in the fungus *Verticillium dahliae* decreased virulence on different host plants [41]. In contrast, the complete deletion of NLPs in the fungal pathogens *Magnaporthe oryzae*, *Mycosphaerella graminicola*, and *Botrytis elliptica* did not reduce their virulence, suggesting that NLP genes of those fungi are dispensable for the fungal pathogen to cause disease [27,42,43]. There is a wide functional diversity of NLPs among plant pathogens that need further exploration.

Several species of the family Botryosphaeriaceae have been classified as human opportunists, suggesting that they possess the molecular machinery that allows infecting both plants and humans [44,45]. Nonetheless, there are no reports describing the contribution of NLPs from those human pathogens by testing on mammalian cells.

We aimed to, for the first time, identify and characterize NLPs genes from *N. parvum* (NprvNeps), in order to infer their role in *N. parvum* pathogenicity.

## 2. Results

### 2.1. Effect of Culture Filtrate of N. parvum on Detached Tomato Leaves

**Figure 1:**
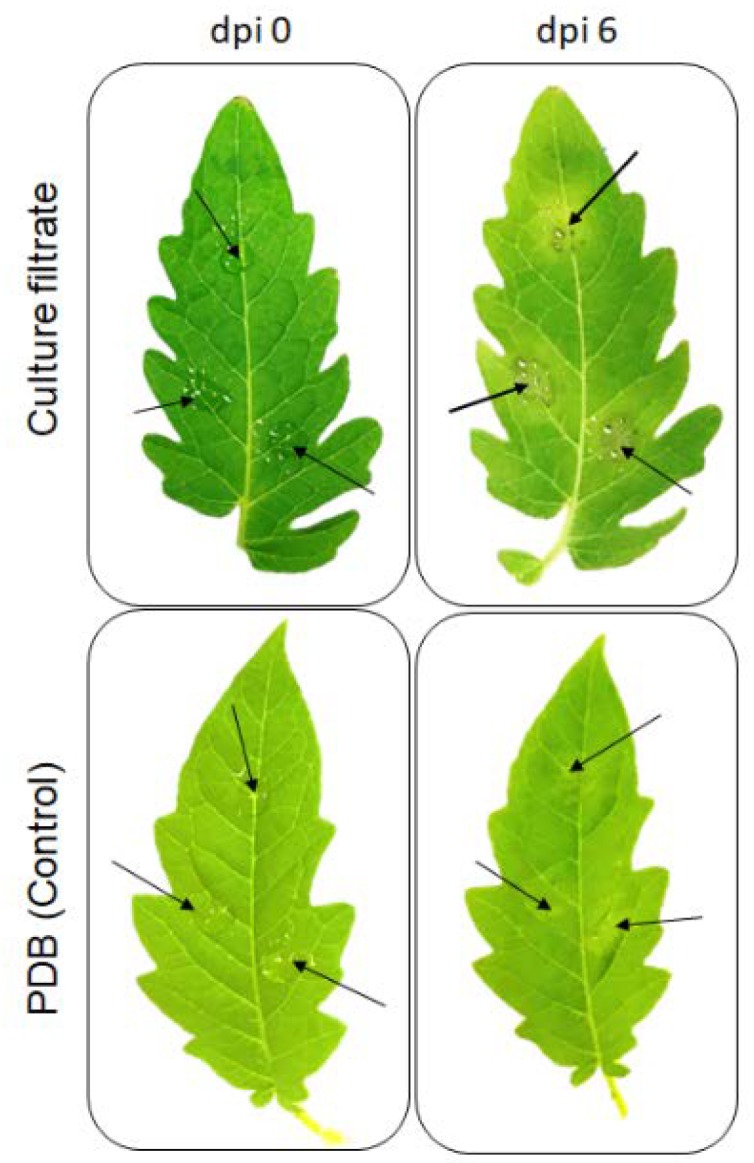
Effect of culture filtrate of *N. parvum* CAA704 on detached tomato leaves after 6 dpi. Potato dextrose broth (PDB) was used as a control. Photographs are representative of three independent experiments, and similar results were obtained in each experiment.

In a preliminary test, we observed that inoculation of 10-day-old *N. parvum* CAA704 culture filtrate into tomato leaves induced necrosis on the treated leaf (Figure 1). The first symptoms were observed approximately at 2 days post-inoculation (dpi) with yellowing around inoculated sites. As time passed, these lesions gradually became irregular, dark brown, surrounded by a yellow halo, at 6 dpi. In contrast, no symptoms were observed in the control leaves.

### 2.2. NprvNep Proteins’ Sequence Analysis

Six NLP genes containing a necrosis-inducing phytophthora protein domain (NPP1) were identified in the genome of *N. parvum* UCRNP2 [46] and named NprvNep 1-6 (Table S1). Of these NLPs, the size of the predicted protein NprvNep6 (163 aa) was dramatically short due to a truncation detected at the N-terminus of the protein. In addition, one of two conserved cysteine crucial for the stability of the protein is absent, leading to a non-functional product. Therefore, NprvNep6 was not further analyzed. Five genes (NprvNep1, NprvNep2, NprvNep3, NprvNep4, and NprvNep5) were selected for functional analysis.

**Figure 2:**
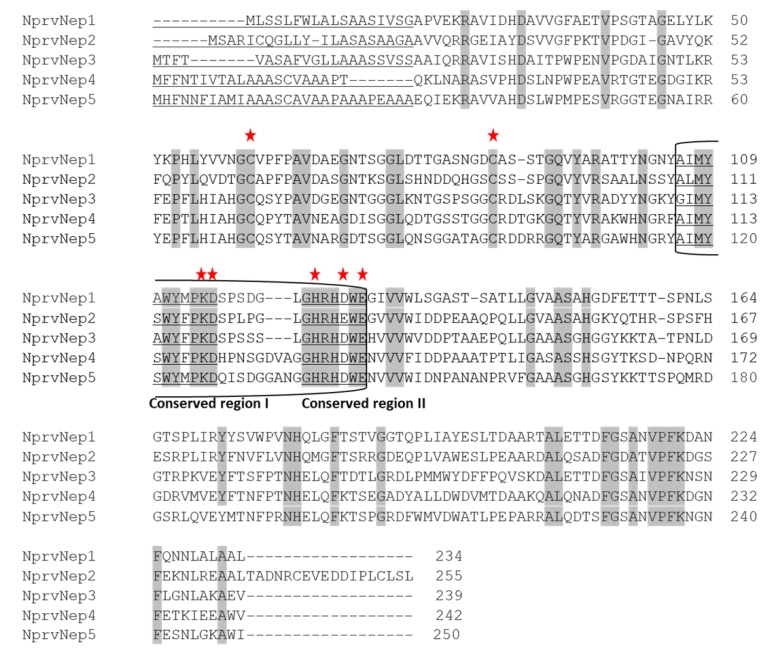
Alignment of the predicted amino acid sequences of the *N. parvum* NLPs proteins. Conserved amino acid residues are shaded grey. Asterisks indicate residues crucial for NLP activity [20,32]. The nlp24 peptide consisting of conserved region I (11-aa) and conserved region II (the heptapeptide GHRHDWE motif) [31,33] is denoted by a box. Signal peptide, conserved regions I and II are underlined.

The conservation of these key residues among *N. parvum* NLPs was assessed (Figure 2). The presence of two conserved cysteines in all NprvNep predicted sequences, confirms that the five NprvNep proteins belong to type 1. Conservation of the 24-aa peptide was detected in all NprvNeps, with some variations. The first four residues (AIMY) of the conserved region I are conserved in NprvNep1, 4, and 5, but variable in NprvNep2 and 3. The conserved region II, GHRHDWE, was also present in NprvNep 1-5. A mutation on heptapeptide sequence was noticed only on NprvNep2, from GHRHDWE to GHRHEWE (Figure 2). All NprvNep proteins have a signal peptide consisting of 18 to 28 amino acid residues, targeting them for secretion.

### 2.3. Cloning, Expression and Purification of NprvNep Proteins

The NprvNep genes were successfully amplified from *N. parvum* genomic DNA. Amplicon sequencing indicated that all NprvNep genes contain one intron, except for NprvNep5 which has 2 introns (Figure S1). The cDNAs of NprvNeps 1-5, without the signal peptide, were successfully cloned in a pET SUMO vector. This vector allows expression of a recombinant protein with an N-terminal peptide containing the hexahistidine tag (H6-SUMO tag) fused to a SUMO protein.

**Figure 3:**
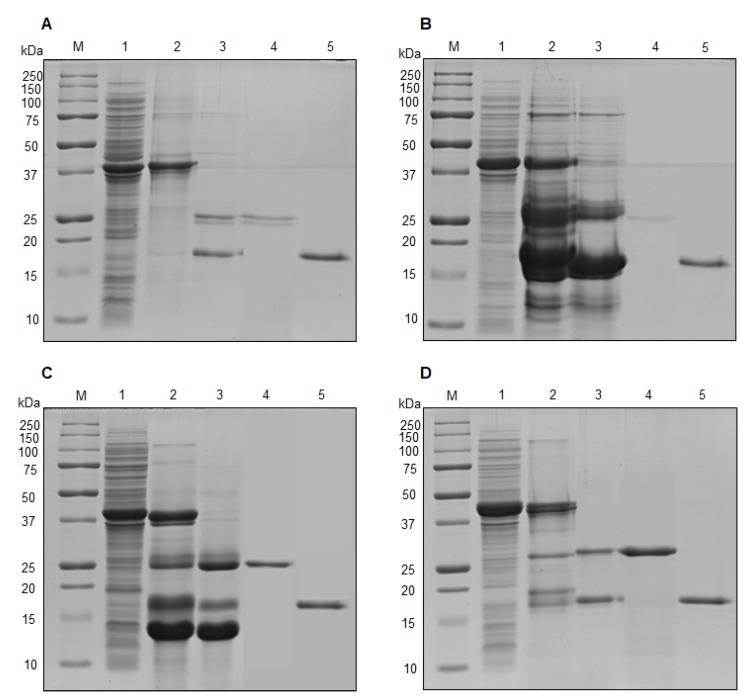
Protein expression and purification of recombinant NprvNeps. SDS-PAGE analysis of (**A**) NprvNep1 protein; (**B**) NprvNep2 protein; (**C**) NprvNep3 protein; and (**D**) NprvNep4 protein. Lanes correspond to: (M) Molecular weight marker; (1) Crude extract of lysed cells 24 h after IPTG induction; (2) H6-SUMO fusion protein after HisTrap purification; (3) SUMO protease-treated fusion protein (4) purified recombinant NprvNep protein (after removal of H6-SUMO tag by HisTrap column); (5) purified 6×His-SUMO by HisTrap column.

Recombinant his-tagged NprvNep1-4 proteins were successfully overexpressed in *E. coli* BL21 (DE3) and the optimum induction conditions for them were obtained with 1 mM IPTG at 30 °C for 24 h (Figure 3, lane 1). The overexpression of NprvNep5 was not possible in this expression system, probably due to very high associated toxicity.

Each fusion protein was purified from the soluble fraction of a cell lysate by affinity binding of the H6 tag to nickel beads, under nondenaturing conditions (Figure 3, lane 2). The N-terminal H6-SUMO tag was removed by SUMO protease (Figure 3, lane 3). Cleavage products were successfully purified as pure NprvNep proteins (Figure 3, lane 4) while H6-SUMO tag remained bound to the column and eluted later at higher concentration of imidazole (Figure 3, lane 5). SDS-PAGE analysis of pure proteins clearly shows a single band for purified protein with the expected molecular weight of the NprvNep proteins.

### 2.4. Activity of NprvNep Proteins—Toxicity to Tomato Leaves

The necrosis-inducing activity of the recombinant NprvNep proteins to tomato leaves was tested. Leaves from young tomato plants were excised and inoculated with each protein (1 to 20 µM). Symptoms were measured for up to 8 days for presence and extent of local necrosis lesions.

**Figure 4:**
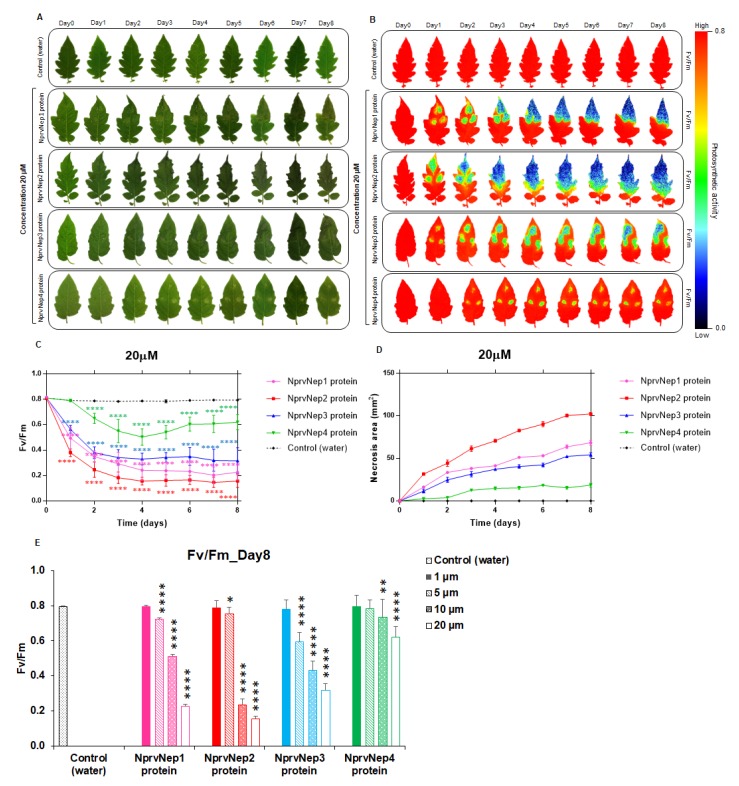
Effect of recombinant NprvNeps on detached tomato leaves. Photographs of tomato leaves inoculated with 20 µM recombinant NprvNeps (**A**), chlorophyll fluorescence (**B**), evolution of F_v_/F_m_ (**C**), evolution of the necrosis area (**D**) and comparison of F_v_/F_m_ at 0 and 8 dpi (**E**). Ultra-pure water was used as a control. The color scale bar indicates the F_v_/F_m_ intensity of the leaf pixels given in false colors from high (red) to low (black) values. All measurements were performed in biological triplicates and error bars show the standard deviation. Two-way ANOVA, followed by a Dunnett’s multiple comparison test (**C**) and a Tukey multiple comparison test (**E**) was used to determine the statistical significance of phytotoxicity of each protein within the same concentration against the control (C) (* *p* < 0.05, ** *p* < 0.01, **** *p* < 0.0001).

No symptoms were observed in controls during the time of the experiment (Figure 4A). None of the recombinant NprvNeps induced visible effects on detached tomato leaves at 1 µM after 8 dpi (Figure S2A). At 5 µM, NprvNep3 induced a distinct white necrosis (at 2 dpi, Figure S3A) that gradually expanded and turned brown. At the same concentration (5 µM), NprvNep2 induced very small white areas. NprvNep1 induced a small discoloration around the punctured area in the inoculated site at 4 dpi, and necrosis was detected at 5 dpi (Figure S3A). NprvNep4 induced no visible symptoms until 5 dpi, when very small necrotic lesions were detected (Figure S3A).

At 10 µM, necrosis symptoms were visible at 1 dpi for NprvNep1, 2, and 3. These lesions expanded over time, especially in the case of NprvNep2-induced lesions. NprvNep4 induced chlorotic areas at 3 dpi that did not evolve until the end of the experiment (Figure S4A).

Tomato leaves treated with 20 µM of recombinant NprvNeps (Figure 4A) developed necrosis at 1 dpi for NprvNeps 1, 2, and 3, and at 2 dpi for NprvNep4. The leaves inoculated with NprvNep2 were severely damaged: the necrotic area expanded through the leaves that died at 3 dpi. Similar symptoms were observed for NprvNep1 and 2, but with milder severity. NprvNep4 induced visually detectable necrosis symptoms only at this concentration (20 µM).

### 2.5. Activity of NprvNep Proteins—Effect of NprvNep Proteins on Chlorophyll Fluorescence

The visual symptoms caused by the inoculation of recombinant NprvNeps into tomato leaves were analyzed by chlorophyll fluorescence imaging (Figure 4B,C). The average F_v_/F_m_ values for control leaves were consistently above 0.79 throughout the experiment time course (Figures S2–S4C and Figure 4C). As expected, F_v_/F_m_ values for leaves inoculated with 1 µM NprvNep proteins showed no significant differences in relation to the control leaves (Figure S2). F_v_/F_m_ of leaves exposed to 5 µM of recombinant NprvNep 4 (at 8 dpi) revealed no significant difference to healthy plants (Figure S3C). For recombinant NprvNeps 1 and 2 a slight, but significant, decline in F_v_/F_m_ values from 0.81 ± 0.003 and 0.82 ± 0.005 to 0.72 ± 0.04 (*p* < 0.0001) and 0.75 ± 0.04 (*p* < 0.001), respectively, were measured at 8 dpi (Figure S3C). However, under 5 µM, NprvNep3 induced a slight decrease in F_v_/F_m_ (0.70 ± 0.03, *p* < 0.0001) at 2 dpi that steadily decreased until the end of the experiment: 0.63 ± 0.04 (*p* < 0.0001) at 3 dpi, and 0.60 ± 0.03 at 8 dpi (*p* < 0.0001) (Figure S3C). These F_v_/F_m_ values results are consistent with the symptoms development.

At 10 µM (1 dpi), recombinant NprvNeps 1-3 induced a significant decrease in F_v_/F_m_: from 0.82 ± 0.005, 0.81 ± 0.003 and 0.81 ± 0.005 to 0.73 ± 0.03 (*p* < 0.001), 0.55 ± 0.04 (*p* < 0.0001) and 0.63 ± 0.03 (*p* < 0.0001) respectively. In contrast, NprvNep4 induced no significant changes (Figure S4C). After 8 dpi, all leaves exhibited significant changes in the values of F_v_/F_m_ (Figure S4C): F_v_/F_m_ of 0.23 ± 0.05, 0.43 ± 0.05, 0.51 ± 0.05 and 0.73 ± 0.04 for leaves challenged with NprvNep 2 (*p* < 0.0001), NprvNep 1 (*p* < 0.0001), NprvNep 3 (*p* < 0.0001), and NprvNep4 (*p* < 0.001), respectively (Figure S4C).

A rapid decrease in F_v_/F_m_ associated with the inoculation of a higher concentration of recombinant NprvNep proteins (20 µM) was observed (Figure 4C). The F_v_/F_m_ values for leaves inoculated with NprvNeps 1, 2 and 3 proteins dramatically decreased within 1 dpi from 0.82 ± 0.005, 0.81 ± 0.005 and 0.81 ± 0.0 to 0.49 ± 0.09 (*p* < 0.0001), 0.38 ± 0.03 (*p* < 0.0001) and 0.56 ± 0.04 (*p* < 0.0001), respectively (Figure 4C). These values were consistent with the visible severe necrosis symptoms observed after the inoculation of NprvNep proteins on tomato leaves at 1dpi (Figure 4A). At the end of the experiment (8 dpi), leaves were severely damaged by recombinant NprvNeps leading to F_v_/F_m_ values of 0.16 ± 0.05, 0.23 ± 0.06, 0.32 ± 0.1 and 0.62 ± 0.06 (NprvNeps 2, 1, 3 and 4, respectively, as shown in Figure 4C).

Necrosis area induced by recombinant NprvNep proteins (10 and 20 µM) was determined (Figure S4D and Figure 4D). All tomato leaves treated with NprvNep1, 2, and 3 at 20 µM showed large necrosis areas (68.3 ± 3.06, 102.3 ± 1.87 mm^2^ and 54.3 ± 2.28, respectively) at 8 dpi (Figure 4D). In contrast, inoculation with NprvNep4 resulted in the smallest lesions (18.6 ± 2.26 mm^2^) (Figure 4D). Interestingly, our data indicate that necrosis area correlates well with a decrease in the Fv/Fm values (Figure S5), mainly for NprvNep 1, 2 and 3 (high necrosis area/ low F_v_/F_m_ values). For very low necrosis areas (initial symptom development), F_v_/F_m_ seems to be a more sensitive parameter.

A summary of the comparison between F_v_/F_m_ values for leaves treated with water and different concentration of NprvNep1-4 proteins at 0 dpi and 8 dpi is represented in Figure 4E, discussed in detail previously. We also compared their symptom development at 0 dpi and 8 dpi by F_v_/F_m_ images, which is shown in Figure S6B.

### 2.6. Activity of NprvNep Proteins—Toxicity to Vero Cells

**Figure 5:**
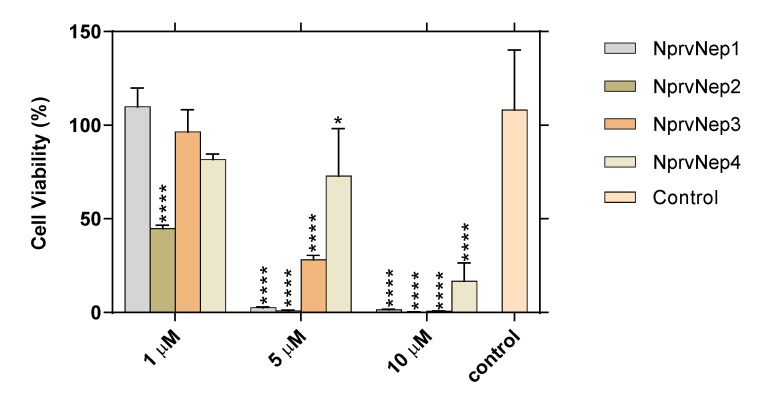
Cytotoxicity of pure recombinant NprvNep proteins to Vero cells. Pure recombinant NprvNep1, 2, 3, and 4 were tested on Vero cells at three different concentrations (1, 5, and 10 µM). Two-way ANOVA, followed by a Tukey multiple comparison test was used to determine the statistical significance of cytotoxicity of each protein within the same concentration against the control (* *p* < 0.05, **** *p* < 0.0001). Data is presented as average ± standard error.

We investigated the cytotoxic effect of recombinant NprvNeps to mammalian Vero cell line (Figure 5). All pure recombinant proteins significantly decreased Vero cells’ viability at all concentrations tested, except for NprvNep1, NprvNep3, and NprvNep4 at 1 µM (Figure 5).

Cell mortality increased with increasing protein concentration, leading to the loss of more than 98% of cell viability for NprvNeps1, 2 and 3 at 10 µM. NprvNep2 was the most toxic, being able to cause 55% mortality at 1 µM. NprvNep4 induced weaker, but, nevertheless, still significant toxic effects for mammalian cells.

## 3. Discussion

Aiming to understand the potential influence of NLP proteins from *N. parvum* in its virulence, we cloned and expressed four genes. In addition, knowing that the family Botryosphaeriaceae comprises several opportunistic human pathogens, we hypothesized that NprvNep proteins could be toxic not only for plants, but also for mammalian cells. In recent decades, various studies have shown that *N. parvum* virulence is associated with the production of a variety of compounds in its hosts as well as in artificial media, such as secreted proteins and phytotoxins [10,11,12,13,47]. However, very little is known about the effector proteins that contribute to the virulence of *N. parvum*, as well as other Botryosphaeriaceae virulence. Cobos et al. [13] reported the presence of three necrosis and ethylene inducing proteins in the secretome of *D. seriata*. Later, the same authors have cloned the NLP genes and expressed in *E. coli* BL21 (DE3) and subsequently purified the proteins. The pure recombinant proteins were phytotoxic to *Vitis* cell culture [48]. We hypothesized that homologous proteins are expressed by *N. parvum* and analyzed *N. parvum* genome for NLP genes. In fact, the analysis of the genome of *N. parvum* revealed the presence of six NLP genes that were named NprvNep 1-6 (Table S2). The presence of a signal peptide is crucial for NLP-induced necrosis since NLPs’ activity is extracellular [25]. Therefore, proteins [48] lacking signal peptide should not be functional *in vivo*. Furthermore, the formation of at least one disulphide bridge is required for NLPs’ activity [17]. Selected NprvNep genes (1–5) contained both requisites: a signal peptide and two conserved cysteine residues, belonging to the type 1 NLPs [17]. NprvNep6 lacked the signal peptide, one conserved cysteine and the upstream sequence, making this protein unqualified for further analysis. A similar truncated version of NLPs was described for *Verticillium dahliae*, in which a lack of the N-terminal, including the two conserved cysteines, was suggested as being responsible for the absence of necrotic and elicitor activities of VdNLP6 [49].

The 24-aa (nlp24) sequence is a highly central conserved region in NLPs and can trigger plant immune responses [31,33]. Substitutions in the nlp24 region of the fungal VdNLP2 protein lead to, in most cases, loss of cytotoxicity [49]. All NprvNep proteins contain the nlp24 peptide with a variable degeneracy (Figure 2). This peptide has two regions that are strongly conserved in type 1 NLPs: conserved regions I and II (Figure 2). Conserved region I (11 aa) contains relevant residues for the phytotoxic activity of NLP such as D and K (Figure 2) [32]. Conserved region II is a seven–amino-acid motif, GHRHDWE, strongly conserved among NLP of different species. Ottman and co-workers [32] also investigated three amino acid residues in this motif (GHRHDWE) showing that these residues are required for cytotoxicity [32]. These residues are involved in the formation of a cation-binding pocket [32]. The acidic pocket was proposed to interact with polar head groups of membrane lipids, thereby damaging or interacting with the plant cell membrane [50]. Our results show that the heptapeptide motif of NprvNep proteins is strongly conserved. The exception is NprvNep 2 heptapeptide, which has a mutation: aspartic acid (D138) to glutamic acid (E) (Figure 2). Our data suggest that this is a conservative mutation, since NprvNep2 protein exhibits high phytotoxic activity compared to the rest of NprvNep, and that this substitution did not affect the cytotoxicity. We noted that key residues are mostly conserved among NprvNep proteins (Figure 2). However, NprvNep2 exhibiting the D138→E substitution, induced stronger symptoms to the detached tomato leaves. Similarly, two NLP of *Phytophthora capsica* (Pc109174 and Pc118548) with the variable residues at the key sites of the heptapeptide motif showed to be phytotoxic [51]. Furthermore, PsNLP54 lacking a conserved Cys residue can cause necrosis [52]. In contrast, six PsNLPs are not able to trigger necrosis even though they contain all the key residues [52]. These findings indicate that it is not straightforward to predict the phytotoxicity of NLPs from sequence analysis only [52].

Several studies have been performed to test phytotoxic ability of NLP by infiltration of recombinant pure proteins in detached leaves. In 1995, Bailey [16], who was the first to purify NLP proteins from culture filtrate of the plant pathogen *Fusarium oxysporum*, showed that the application of only 50 ng of the protein could induce ethylene and necrosis on cacao leaves [16]. Similarly, infiltration of 2.5 µM of *Phytophthora parasitica* NPP1 into parsley and *Arabidopsis* leaves and 2 µM in tobacco leaves resulted in necrotic lesion formation. However, infiltration of similar and higher concentrations (10 µM) of NLP protein failed to induce necrosis in monocot plants [20]. Purified BcNEP1 (0.04 to 4 μM) and BcNEP2 (0.4 to 40 μM) from the necrotrophic fungus *Botrytis cinerea* caused necrosis in all dicotyledonous plant species tested, but not in monocotyledons [26]. In *Moniliophthora perniciosa*, the causal agent of witches’ broom in *Theobroma cacao*, MpNEP1 and 2 also showed necrosis and ethylene emission after infiltration into tobacco and cacao leaves at 1 µM [39]. However, infiltration of NLPs into detached leaves of plants does not always cause cell death and necrosis symptoms. Purified HaNLP1, 2, and 3 (2–20 µM) from downy mildew pathogen *Hyaloperonospora arabidopsidis*, failed to induce necrosis into leaves of *Arabidopsis thaliana* Col-0 and *Nicotiana tabacum* [53]. Similarly, the sole NLP gene in fungal wheat leaf pathogen *Mycosphaerella graminicola* induced necrotic cell death and defense-related genes after infiltration into *Arabidopsis* leaves at 2 µM, but not in the same concentration in leaves of a susceptible wheat genotype [43].

NLPs are best known to cause necrosis, cell death and wilting symptoms on plants [20,54]. To analyse whether the NLPs from *N. parvum* can cause necrosis, we evaluated the effect of heterologously-expressed NprvNep1-4 proteins, *in vitro*, on detached tomato leaves and on a mammalian cell line. The lack of overexpression of the NprvNep5 is likely associated with the high toxicity of NprvNep5 for *E. coli* cells. Even after several attempts (including transformation of NprvNep5 in a more resistant strain of *E. coli*- BL21 (DE3) pLysS competent cells; the addition of glucose to culture medium; among others), NprvNep expression was not attained (data not shown). We were able to confirm that NprvNep genes encode biologically active proteins able to cause necrosis in tomato leaves, in a dose-dependent manner. The fact that the same gene family of NLP could have distinct phytotoxic activities was also observed in the case of BcNEPs from *B. cinerea*. Both BcNEP1 and BcNEP2 induced necrosis in all tested leaves of dicot plants, but BcNEP2-induced symptoms were consistently less severe than those induced by BcNEP1 [55]. Similarly, phytotoxic activity of NLPs from a tomato-pathogenic *V. dahliae* strain were distinct, whereas only two of the seven NLP displayed cytotoxic activity in plants [41].

Besides the classic visual observation of symptoms, we assessed the photosystem II (PSII) activity of tomato leaves upon inoculation of the recombinant NprvNeps. Photodamage resulting from biotic or abiotic stress factors was indicated by a reduction in F_v_/F_m_ values, from the typical 0.80 of unstressed leaves. Extensive studies have used F_v_/F_m_ images to follow fungal infections such as *Colletotrichum lindemuthianum* in *P. vulgaris* [56], *B. cinerea* in *S. lycopersicum* [57], *Puccina polysora* in *Zea mays* [58] and *Colletotrichum orbiculare* in *Nicotiana benthamiana* [59]. Our results showed that the F_v_/F_m_ values of recombinant NprvNep treated leaves exhibited a time and concentration-dependent decrease, representative of the photosynthetic damage induced by NprvNeps.

It is well established that NLPs toxicity is restricted to dicot plants [17,18,26,35]. Nonetheless, bacterial virulence and hemolysis activity of *Vibrio navarrensis* against animal erythrocytes has been related to a genomic region that contained a gene encoding an NLP [30]. Furthermore, type 2 NLPs in animal-related microorganisms such as the coral pathogen *Vibrio coralliilyticus* [60] and the bivalve endosymbiont *Teredinibacter turnerae* [61] have been reported, but additional studies are needed to clarify whether their NLPs are functional and have cytotoxic effects on mammalian cells or not. As far as we are aware, only one report described the activity of NLPs towards other cells like animal, moss, yeast, and lower plants’ cells [25]. The viability of each of these cell types was not significantly reduced by incubation with up to 1 μM NLP. In contrast, at 1 μM, NprvNep 2 induced cell mortality. At higher concentrations, all NprvNeps induced cell mortality in a dose dependent manner. A similar trend of toxicity to the one observed in tomato leaves was observed in Vero cells (the most toxic NprvNep 2 and the least toxic NprvNep 4) suggesting that similar pathways may be used in plants and mammals. In addition, the susceptibility of Vero cells to pure recombinant NprvNeps suggests implications in the ability of *N. parvum* to infect animal/human hosts. This is the first report of animal cell toxicity induced by NLPs proteins, but to rule out a general effect of NLPs on any cell type more tests need to be done with a larger range of cell lines.

## 4. Conclusions

We characterized, for the first time, the NLPs from the plant pathogen, *N. parvum*. Our results showed that four NLP genes in *N. parvum* are functional genes encoding proteins toxic both to plant and mammalian cells, most probably involved in virulence or cell death during infection by *N. parvum*. We also showed that chlorophyll fluorescence imaging can be used to accurately quantify the effect of toxic proteins on plant leaves. In fact, we provided the first monitoring of NLPs effect on detached leaves using the commonly used chlorophyll fluorescence index Fv/Fm.

## 5. Materials and Methods

### 5.1. Fungal Strain and Plant Material

The strain used in this study, *Neofusicoccum parvum* CAA704, was isolated from symptomatic branch of *Eucalyptus globulus* in Portugal by Barradas and co-workers [62]. Fungus was grown on cellophane over Czapek-Dox agar plate at 25 °C for 5 days prior to the inoculations. For liquid growth, a plug (5 mm diameter) was inoculated into a 250 mL flask containing 50 mL Czapeck-Dox broth and incubated at 25 °C for 3 days in an orbital shaker at 150 rpm. Mycelia were harvested by filtration (filter paper), washed with sterile water and immediately frozen with N_2_ (l) prior to DNA and RNA extraction.

The tomato seeds (*Solanum lycopersicum* var. *cerasiforme*) were cultivated in plastic trays filled with vermiculite:peat [2:1 (w/w)] mixture and kept at 25–28 °C (16h light period) in a growth chamber. All seedlings were equally well watered and fertilized weekly (5 mL/L Nutriquisa 5-8-10^®^) and grown for 90 days under the conditions described.

### 5.2. DNA and RNA Extraction and cDNA Synthesis

The extraction of genomic DNA from mycelium (120 mg) was performed as described by Alves et al. [63]. Genomic DNA was used for the amplification of NprvNep genes by PCR using the primers (Table S2) designed from *N. parvum* (UCRNP2) genomic sequence in the NCBI database. RNA was extracted from 50 mg of frozen mycelia with Spectrum™ Plant Total RNA Kit (Sigma-Aldrich, St. Louis, MO, USA) following the manufacturer’s instructions. The quality and quantity of both DNA and RNA were checked by gel electrophoresis (1% agarose gel) and spectrophotometrical assays. A total of 1 μg RNA was treated with DNase l, RNase-free (Thermo scientific, Lisbon, Portugal) and cDNA was synthesized using an NZY First-Strand cDNA Synthesis Kit (NZYTech, Lisbon, Portugal) according to the manufacturer’s instructions.

### 5.3. Cloning, Expression and Purification of Recombinant NprvNeps

The synthesized cDNA was used as a template for amplification of NprvNep1-5 genes using primers (designed from *N. parvum*—UCRNP2 genomic sequence in the NCBI database) described in Table S2, supplementary file. PCR reactions were prepared with 2× Platinum SuperFi PCR Master Mix (Invitrogen, Carlsbad, CA, USA), 10 µM of each primer, and 20 ng of template DNA. The program consisted of an initial step of 30 s at 98 °C, followed by 34 cycles of denaturation at 98 °C for 10 s, annealing at 65 °C for 10 s, and elongation at 72 °C for 30 s. A final extension was performed at 72 °C for 5 min. For each amplification, PCR products were excised and purified from gel using Zymoclean™ Gel DNA Recovery Kit (Zymo Research, Irvine, CA, USA). Purified sequence-verified PCR products were cloned into pET SUMO vector using ChampionTM pET SUMO protein expression system (Invitrogen, CA, USA) according to the manufacturer’s instructions. The One Shot^®^ Mach1™-T1R Chemically Competent *E. coli* (Invitrogen, CA, USA) was transformed with the recombinant plasmid constructions. Transformants were analyzed by restriction analysis to confirm the presence and correct orientation of the insert. Recombinant plasmids were extracted from positive transformants and confirmed by sequencing and propagated in BL21 (DE3) One Shot^®^ Chemically Competent *E. coli* (Invitrogen, Carlsbad, CA, USA) cultured in LB broth supplemented with 50 µg/mL kanamycin. Protein expression was induced with 1 mM isopropyl-1-thio-b-D-galactopyranoside (IPTG), producing proteins with his-tags. Cells were harvested by centrifugation, resuspended in a lysis buffer (20 mM sodium phosphate; 500 mM NaCl; 10 mM imidazole; 2% SDS, pH 7.4) and sonicated. Cell debris was removed by centrifugation (10,000 g for 10 min at 4 °C) and the supernatant filtered (0.20 μm pore size filter, Orange Scientific) and loaded onto a His-Trap FF Ni affinity column (GE Healthcare). The recombinant His6-tagged proteins were eluted in the elution buffer (20 mM sodium phosphate; 500 mM NaCl; 500 mM imidazole, pH 7.4) using an ÄKTA FPLC system (GE Healthcare, Upsala, Sweden). Fractions containing His6-tagged recombinant NprvNeps were pooled and dialysed overnight against ultrapure water at 4 °C in dialysis cellulose membranes (Sigma-Aldrich, Darmstadt, Germany). The desalted recombinant fusion protein solutions were then lyophilized and solubilized in ultrapure water. To remove the 6xHis tag and SUMO protein and generate native recombinant proteins, the purified recombinant fusion proteins were treated with SUMO Protease (Invitrogen) according to the manufacturer’s instructions. Subsequently, recombinant proteins were purified by Ni affinity chromatography (His-Trap FF, GE-Healthcare, Upsala, Sweden). Salts were removed by dialysis against ultrapure water, lyophilized and stored until further analysis. The protein purity was assessed by SDS–PAGE [64] at the various stages of the purification process.

### 5.4. Protein Concentration

Protein concentration was determined using the Pierce™ 660 nm Protein Assay kit (Thermo Scientific, Oeiras, Portugal) according to the manufacturer’s instructions. Bovine Serum Albumin was used as standard. The procedure was performed in triplicate.

### 5.5. Phytotoxic Activity

Recombinant NprvNeps were inoculated into detached leaves of 3-month old tomato plants, which is an important model plant system as well [65], to assess their ability to cause necrosis. Each detached leaf was punctured on 3 spots with a sterile needle and the stem placed in sterile water in a closed Petri dish to avoid dehydration. Twenty µl drops (3 drops per leaf) of four concentrations of the recombinant NprvNep proteins (1, 5, 10, and 20 µM) were placed on the top of the leaves at the punctured spots (three leaves per treatment). Sterile ultrapure water was used as a control. Inoculated leaves were kept at room temperature (22–25 °C). Symptom development was monitored for 8 days after inoculations. The same procedure was used for *Eucalyptus* leaves without success. *Eucalyptus* leaves presented considerable water repellence with the droplets tending to avoid the leaf surface. Results did not have the quality for publication.

### 5.6. Chlorophyll Fluorescence Imaging

*In vivo* chlorophyll fluorescence images of tomato leaves were measured by using a FluorCAM 800MF imaging fluorometer (Photon System Instruments, Brno, Czech Republic), comprising a computer operated control unit (SN-FC800-082; Photon System Instruments) and a CCD camera (CCD381; PSI) with a f1.2 (2.8–6 mm) objective (Eneo, Rödermark, Germany), as described by Serôdio et al. [66]. Images of chlorophyll fluorescence parameters Fo and Fm (dark-level and maximum fluorescence level, respectively), were captured on tomato leaves dark-adapted for 20 min by applying modulated measuring light and saturation pulses (<0.1 and >7500 μmol photons m^−2^ s^−1^, respectively), provided by red LED panels (612 nm emission peak, 40-nm bandwidth). Images (512 × 512 pixels) were processed by defining areas of interest (AOIs) matching the whole area of each leaf, by excluding the non-fluorescent background signal (outside the leave) and the necrosis areas (see below), using the FluorCam7 software (Photon System Instruments, spol. s r.o., Drasov, Czech Republic). Areas of necrosis were quantified by counting the number of pixels of the leave that drawn manually, using the FluorCAM7 software. The values of Fo and Fm were calculated by averaging all pixel values in each AOI [67]. The maximum quantum yield of PSII was calculated as F_v_/F_m_ = (F_m_ − F_o_)/F_m_ [68]. For the production of the images shown in Figure 4B, Figures S2–S4 and S6B, the scale of false colour of F_v_/F_m_ values was normalized between 0.0 and 0.8 to ensure consistency between the different treatments.

### 5.7. Cytotoxicity Assay

*In vitro* cytotoxic effects of recombinant NprvNeps was assessed as described previously [69,70] with slight modifications. A Vero cell line (ECACC 88020401, African Green Monkey kidney), widely used for the assessment of the cytotoxic effects of chemicals, toxins and other substances on mammalian cells at the molecular level [71], was grown and maintained according to Ammerman et al. [71]. Microtiter plates were incubated at 37 °C in 5% CO_2_ for 24 h. Recombinant NprvNeps in PBS were filtered through a sterile 0.2 µm pore size syringe membrane filter and prepared at 1 µM, 5 µM, 10 µM concentrations. All NprvNep dilutions then were added to Vero cells [1:1 (v/v) in DMEM - Dulbecco’s Modified Eagle Medium] and incubated for 20 h. After cell treatment, the medium was removed by aspiration and 50 µL of DMEM with 10% resazurin (0.1 mg/mL in PBS) was directly added to each well. The microtiter plates were incubated at 37 °C in 5% CO_2_ for 3 h. The absorbance was read at 570 and 600 nm in a microtiter plate spectrophotometer (Biotek Synergy, Colmar, France). Phosphate-buffered saline buffer (PBS) was used as control.

### 5.8. Bioinformatics Analysis

Gene database from NCBI was used to detect putative NprvNep genes in the genome of *N. parvum* isolate UCR-NP2. The SignalP 4.1 Server [72] was used to identify the signal peptide sequences. Molecular weight (MW) predictions for the deduced protein were carried out using the Compute pI/Mw tool (Lausanne, Switzerland, www.expasy.org/tools/protparam.html).

### 5.9. Statistical Analysis

Two-way analysis of variance (ANOVA) followed by a Tukey multiple comparison test was used to determine the statistical significance of cytotoxicity of each recombinant NprvNeps within the same concentration against the control (* *p* < 0.05, ** *p* < 0.01, *** *p* < 0.001, **** *p* < 0.0001). Differences between Fv/Fm among the different experiences were tested using a two-way ANOVA, followed by the Dunnett’s multiple comparison test and Tukey multiple comparison to determine the statistical significance of phytotoxicity of each protein within the same concentration against the control (* *p* < 0.05, ** *p* < 0.01, *** *p* < 0.001, **** *p* < 0.0001). All the analyses were performed with GraphPad Prism v.7 (GraphPad Software, version 8, Inc., La Jolla, CA, USA). Data are shown as the average of three independent replicates of each condition.

## Supplementary Materials

The following are available online at https://www.mdpi.com/2072-6651/12/4/235/s1, Figure S1: Alignment of the DNA and cDNA sequence of *Neofusicoccum parvum* NLPs, Figure S2: Effect of 1 µM recombinant NprvNeps on detached tomato leaves symptoms development, Figure S3: Effect of 5 µM recombinant NprvNeps on detached tomato leaves symptoms development, Figure S4: Effect of 10 µM recombinant NprvNeps on detached tomato leaves symptoms development, Figure S5: Scatter plot of necrosis area vs. F_v_/F_m_ values for 8 days, Figure S6: Toxicity of recombinant NprvNeps to detached tomato leaves evaluated by chlorophyll fluorescence, Table S1: Primers used for cloning and amplification, Table S2: The data of 6 NprNep genes.
