# Supplementary material for: Toxicity of Recombinant Necrosis and Ethylene-Inducing Proteins (NLPs) from Neofusicoccum parvum
Source: Toxins (Basel). 2020 Apr 7;12(4):235. doi: 10.3390/toxins12040235 (PMC7232490; doi:10.3390/toxins12040235)
Supplement: Supplementary file 1 [file toxins-12-00235-s001.pdf]

Supplementary Materials: Toxicity of Recombinant Necrosis and Ethylene-Inducing Proteins (NLPs) from *Neofusicoccum parvum*

Forough Nazar Pour, Rebeca Cobos, Juan José Rubio Coque, João Serôdio, Artur Alves, Carina Félix, Vanessa Ferreira, Ana Cristina Esteves, Ana Sofia Duarte

A

```
DNANprvNep1    ATGCTGTCTTCCCTCTTCTGGCTTGCGCTCTCCGCTGCGAGCATTGTTTCAGGCGCCCCG 60
cDNANEP1       ATGCTGTCTTCCCTCTTCTGGCTTGCGCTCTCCGCTGCGAGCATTGTTTCAGGCGCCCCG 60

 DNANprvNep1   GTCGAGAAGCGCGCCGTCATTGATCATGATGCCGTTGTGGGCTTTGCCGAGACGGTCCCC 120
cDNANprvNep1   GTCGAGAAGCGCGCCGTCATTGATCATGATGCCGTTGTGGGCTTTGCCGAGACGGTCCCC 120

 DNANprvNep1   AGCGGCACGGCCGGCGAGCTGTACCTGAAGTACAAGCCGCACTTGTATGTCGTGAACGGC 180
cDNANprvNep1   AGCGGCACGGCCGGCGAGCTGTACCTGAAGTACAAGCCGCACTTGTATGTCGTGAACGGC 180

 DNANprvNep1   TGCGTGCCGTTCCCAGCAGTGGATGCCGAGGGCAACACGAGCTTAGTCTTCCCTCTTATT 240
cDNANprvNep1   TGCGTGCCGTTCCCAGCAGTGGATGCCGAGGGCAACACGAGCG----------------- 223

 DNANprvNep1   TCTGCCGACGACCATCTCGCTCACTCCCTTCCCAGCGGCGGCCTCGACACCACCGGAGCC 300
cDNANprvNep1   -------------------------------------GCGGCCTCGACACCACCGGAGCC 246

 DNANprvNep1   TCCAACGGCGACTGCGCCAGCAGCACCGGCCAGGTCTACGCGCGCGCCACCACCTACAAC 360
cDNANprvNep1   TCCAACGGCGACTGCGCCAGCAGCACCGGCCAGGTCTACGCGCGCGCCACCACCTACAAC 306

 DNANprvNep1   GGCAACTACGCCATCATGTACGCGTGGTACATGCCCAAGGACTCGCCGTCGGACGGGCTG 420
cDNANprvNep1   GGCAACTACGCCATCATGTACGCGTGGTACATGCCCAAGGACTCGCCGTCGGACGGGCTG 366

 DNANprvNep1   GGCCACCGCCACGACTGGGAGGGCATCGTCGTCTGGCTGTCGGGCGCCTCCACCTCCGCC 480
cDNANprvNep1   GGCCACCGCCACGACTGGGAGGGCATCGTCGTCTGGCTGTCGGGCGCCTCCACCTCCGCC 426

 DNANprvNep1   ACCCTGCTCGGCGTCGCCGCCTCCGCCCACGGCGACTTCGAAACCACCACCAGCCCCAAC 540
cDNANprvNep1   ACCCTGCTCGGCGTCGCCGCCTCCGCCCACGGCGACTTCGAAACCACCACCAGCCCCAAC 486

 DNANprvNep1   CTTAGCGGCACCAGCCCTCTCATCCGCTACTACAGCGTCTGGCCCGTCAACCACCAGCTC 600
cDNANprvNep1   CTTAGCGGCACCAGCCCTCTCATCCGCTACTACAGCGTCTGGCCCGTCAACCACCAGCTC 546

 DNANprvNep1   GGCTTCACCAGCACGGTCGGCGGCACGCAGCCGCTCATTGCGTACGAGAGCTTGACGGAT 660
cDNANprvNep1   GGCTTCACCAGCACGGTCGGCGGCACGCAGCCGCTCATTGCGTACGAGAGCTTGACGGAT 606

 DNANprvNep1   GCGGCGAGGACGGCGTTGGAGACTACGGATTTCGGCAGCGCGAATGTGCCGTTCAAGGAT 720
cDNANprvNep1   GCGGCGAGGACGGCGTTGGAGACTACGGATTTCGGCAGCGCGAATGTGCCGTTCAAGGAT 666

 DNANprvNep1   GCGAACTTCCAAAACAACCTAGCGTTAGCGGCGTTGTAG   759
cDNANprvNep1   GCGAACTTCCAAAACAACCTAGCGTTAGCGGCGTTGTAG   705
```

B

```
DNANprvNep2     ATGTCGGCCCGTATCTGCCAAGGACTCCTTTACATTCTTGCCAGTGCCAGCGCAGCCGGT 60
cDNANprvNep2    ATGTCGGCCCGTATCTGCCAAGGACTCCTTTACATTCTTGCCAGTGCCAGCGCAGCCGGT 60

DNANprvNep2     GCTGCGGTTGTTCAACGCCGCGGCGAGATTGCCTATGATTCCGTTGTTGGCTTCCCCAAA 120
cDNANprvNep2    GCTGCGGTTGTTCAACGCCGCGGCGAGATTGCCTATGATTCCGTTGTTGGCTTCCCCAAA 120

DNANprvNep2     ACAGTTCCAGATGGGATTGGTGCCGTCTACCAGAAGTTCCAGCCCTACCTGCAGGTGGAC 180
cDNANprvNep2    ACAGTTCCAGATGGGATTGGTGCCGTCTACCAGAAGTTCCAGCCCTACCTGCAGGTGGAC 180

DNANprvNep2     ACGGGCTGTGCTCCTTTCCCGGCTGTGGATGCCTCAGGAAACACGAACTGAGTGCCACTG 240
cDNANprvNep2    ACGGGCTGTGCTCCTTTCCCGGCTGTGGATGCCTCAGGAAACACGAACTC---------- 230

DNANprvNep2     AAACCAAACTCCGCCGACACCAATAACAACCCATATCTCCGCAGCTCCGGCCTCAGCCAC 300
cDNANprvNep2    -----------------------------------------------CGGCCTCAGCCAC 243

DNANprvNep2     AACGACGACCAGCACGGCAGTTGCTCCAGCAGCCCCGGCCAAGTCTACGTCCGCTCCGCC 360
cDNANprvNep2    AACGACGACCAGCACGGCAGTTGCTCCAGCAGCCCCGGCCAAGTCTACGTCCGCTCCGCC 303

DNANprvNep2     GCCCTCAACTCCAGCTACGCGCTGATGTACTCGTGGTACTTTCCCAAGGACTCGCCGCTC 420
cDNANprvNep2    GCCCTCAACTCCAGCTACGCGCTGATGTACTCGTGGTACTTTCCCAAGGACTCGCCGCTC 363

DNANprvNep2     CCCGGCCTCGGGCACCGGCACGAGTGGGAGGGCGTCGTCGTCTGGATCGACGACCCCGAG 480
cDNANprvNep2    CCCGGCCTCGGGCACCGGCACGAGTGGGAGGGCGTCGTCGTCTGGATCGACGACCCCGAG 423

DNANprvNep2     GCCGCCCAGCCGCAGCTGCTCGGCGTGGCGGCGTCCGCCCATGGCAAGTACCAGACCCAC 540
cDNANprvNep2    GCCGCCCAGCCGCAGCTGCTCGGCGTGGCGGCGTCCGCCCATGGCAAGTACCAGACCCAC 483

DNANprvNep2     AGGAGCCCGAGCTTTCACGAGTCCAGACCGCTGATCAGGTACTTCAACGTTTTCTTGGTC 600
cDNANprvNep2    AGGAGCCCGAGCTTTCACGAGTCCAGACCGCTGATCAGGTACTTCAACGTTTTCTTGGTC 543

DNANprvNep2     AACCATCAGATGGGCTTCACGAGCAGGCGGGGCGACGAGCAGCCGTTGGTGGCGTGGGAG 660
cDNANprvNep2    AACCATCAGATGGGCTTCACGAGCAGGCGGGGCGACGAGCAGCCGTTGGTGGCGTGGGAG 603

DNANprvNep2     AGCCTGCCGGAGGCGGCGAGGGATGCGCTGCAGAGCGCGGACTTTGGGGATGCGACTGTG 720
cDNANprvNep2    AGCCTGCCGGAGGCGGCGAGGGATGCGCTGCAGAGCGCGGACTTTGGGGATGCGACTGTG 663

DNANprvNep2     CCGTTTAAGGACGGGAGTTTCGAGAAGAATCTGCGCGAGGCTGCGTTGACGGCGGACAAC 780
cDNANprvNep2    CCGTTTAAGGACGGGAGTTTCGAGAAGAATCTGCGCGAGGCTGCGTTGACGGCGGACAAC 723

DNANprvNep2     AGATGTGAGGTGGAGGACGACATACCCCTGTGCCTGTCGTTGTGA 825
cDNANprvNep2    AGATGTGAGGTGGAGGACGACATACCCCTGTGCCTGTCGTTGTGA 768
```

C

```
DNANprvNep3     ATGACTTTCACTGTTGCCTCTGCTTTCGTTGGCCTCCTGGCCGCAGCCAGCTCCGTCAGC 60
CDNANprvNep3    ATGACTTTCACTGTTGCCTCTGCTTTCGTTGGCCTCCTGGCCGCAGCCAGCTCCGTCAGC 60

DNANprvNep3     AGTGCTGCCATCCAACGCCGCGCAGTCATTTCCCACGACGCCATCACCCCCTGGCCCGAG 120
CDNANprvNep3    AGTGCTGCCATCCAACGCCGCGCAGTCATTTCCCACGACGCCATCACCCCCTGGCCCGAG 120

DNANprvNep3     AACGTCCCCGGCGATGCCATTGGCAACACGTTGAAGAGATTCGAGCCGTTCCTGCACATC 180
CDNANprvNep3    AACGTCCCCGGCGATGCCATTGGCAACACGTTGAAGAGATTCGAGCCGTTCCTGCACATC 180

DNANprvNep3     GCCCACGGCTGCCAATCCTACCCTGCCGTCGACGGCGAGGGCAACACCGGCTGAGCCGCA 240
CDNANprvNep3    GCCCACGGCTGCCAATCCTACCCTGCCGTCGACGGCGAGGGCAACACCGGC--------- 231

DNANprvNep3     AGCCAACTCCAGCCGCAGCCGGCCACGACACTGACGCGGCCATCTCGCAGCGGCGGCCTG 300
CDNANprvNep3    ---------------------------------------------------GGCGGCCTG 240

DNANprvNep3     AAGAACACAGGCAGCCCGTCGGGCGGGTGCCGCGACCTCTCCAAGGGCCAGACGTACGTG 360
CDNANprvNep3    AAGAACACAGGCAGCCCGTCGGGCGGGTGCCGCGACCTCTCCAAGGGCCAGACGTACGTG 300

DNANprvNep3     CGGGCCGACTACTACAACGGCAAGTACGGCATCATGTACGCGTGGTACTTCCCCAAGGAC 420
CDNANprvNep3    CGGGCCGACTACTACAACGGCAAGTACGGCATCATGTACGCGTGGTACTTCCCCAAGGAC 360

DNANprvNep3     TCGCCGTCGTCGTCGCTGGGCCACCGGCACGACTGGGAGCACGTCGTCGTCTGGGTCGAC 480
CDNANprvNep3    TCGCCGTCGTCGTCGCTGGGCCACCGGCACGACTGGGAGCACGTCGTCGTCTGGGTCGAC 420

DNANprvNep3     GACCCCACCGCCGCCGAGCCACAGCTGCTGGGCGCCGCCGCCTCCGGCCACGGCGGCTAC 540
CDNANprvNep3    GACCCCACCGCCGCCGAGCCACAGCTGCTGGGCGCCGCCGCCTCCGGCCACGGCGGCTAC 480

DNANprvNep3     AAGAAGACGGCCACCCCGAACCTGGACGGCACGCGCCCGAAGGTCGAGTACTTCACCAGC 600
CDNANprvNep3    AAGAAGACGGCCACCCCGAACCTGGACGGCACGCGCCCGAAGGTCGAGTACTTCACCAGC 540

DNANprvNep3     TTCCCCACCAACCACGAGCTGCAGTTCACCGACACCCTCGGCCGCGACCTGCCCATGATG 660
CDNANprvNep3    TTCCCCACCAACCACGAGCTGCAGTTCACCGACACCCTCGGCCGCGACCTGCCCATGATG 600

DNANprvNep3     TGGTACGACTTCTTCCCGCAGGTGAGCAAGGACGCGCTGGAGACCACGGATTTCGGCAGC 720
CDNANprvNep3    TGGTACGACTTCTTCCCGCAGGTGAGCAAGGACGCGCTGGAGACCACGGATTTCGGCAGC 660

DNANprvNep3     GCAATCGTGCCGTTTAAGAACTCGAACTTCTTGGGCAACCTCGCCAAGGCTGAGGTCTGA 780
CDNANprvNep3    GCAATCGTGCCGTTTAAGAACTCGAACTTCTTGGGCAACCTCGCCAAGGCTGAGGTCTGA 720
```

D

```
DNANprvNep4     ATGTTCTTCAACACTATTGTTACCGCTCTCGCTGCTGCTTCCTGCGTTGCAGCTGCTCCC 60
CDNANprvNep4    ATGTTCTTCAACACTATTGTTACCGCTCTCGCTGCTGCTTCCTGCGTTGCAGCTGCTCCC 60

DNANprvNep4     ACGCAGAAGCTGAACGCTCGTGCAAGCGTCCCCCACGACTCGCTCAACCCGTGGCCTGAA 120
CDNANprvNep4    ACGCAGAAGCTGAACGCTCGTGCAAGCGTCCCCCACGACTCGCTCAACCCGTGGCCTGAA 120

DNANprvNep4     GCTGTCAGGACCGGCACTGAGGGTGACGGCATCAAGAGGTTTGAGCCGACTCTCCACATT 180
CDNANprvNep4    GCTGTCAGGACCGGCACTGAGGGTGACGGCATCAAGAGGTTTGAGCCGACTCTCCACATT 180

DNANprvNep4     GCCCATGGCTGCCAGCCGTACACTGCCGTCAACGAAGCCGGCGACATCAGGTTAGTGCCC 240
CDNANprvNep4    GCCCATGGCTGCCAGCCGTACACTGCCGTCAACGAAGCCGGCGACATCAG---------- 230

DNANprvNep4     ATGCTCTCGATCTCCCCTTAGCCCACTAACACACAACACAGCGGCGGCCTCCAAGACACC 300
CDNANprvNep4    -----------------------------------------CGGCGGCCTCCAAGACACC 249

DNANprvNep4     GGCAGCTCCACCGGCGGCTGCAGGGACACCGGCAAGGGCCAGACCTACGTCCGGGCCAAG 360
CDNANprvNep4    GGCAGCTCCACCGGCGGCTGCAGGGACACCGGCAAGGGCCAGACCTACGTCCGGGCCAAG 309

DNANprvNep4     TGGCACAACGGCCGCTTCGCCATCATGTACTCGTGGTACTTCCCGAAGGACCACCCCAAC 420
CDNANprvNep4    TGGCACAACGGCCGCTTCGCCATCATGTACTCGTGGTACTTCCCGAAGGACCACCCCAAC 369

DNANprvNep4     AGCGGCGACGTGGCCGGCGGCCACCGCCACGACTGGGAGAACGTCGTCGTCTTCATCGAC 480
CDNANprvNep4    AGCGGCGACGTGGCCGGCGGCCACCGCCACGACTGGGAGAACGTCGTCGTCTTCATCGAC 429

DNANprvNep4     GACCCGGCCGCCGCCACCCCGACGCTGATCGGCGCCTCGGCATCCAGCCACAGCGGCTAC 540
CDNANprvNep4    GACCCGGCCGCCGCCACCCCGACGCTGATCGGCGCCTCGGCATCCAGCCACAGCGGCTAC 489

DNANprvNep4     ACCAAGAGCGACAACCCCCAGCGCAACGGCGACCGCGTCATGGTCGAGTACTTCACCAAC 600
CDNANprvNep4    ACCAAGAGCGACAACCCCCAGCGCAACGGCGACCGCGTCATGGTCGAGTACTTCACCAAC 549

DNANprvNep4     TTCCCCACCAACCACGAGCTGCAGTTCAAGACCAGCGAGGGCGCCGACTACGCCCTGCTC 660
CDNANprvNep4    TTCCCCACCAACCACGAGCTGCAGTTCAAGACCAGCGAGGGCGCCGACTACGCCCTGCTC 609

DNANprvNep4     GACTGGGACGTTATGACCGACGCTGCCAAGCAGGCCCTCCAGAATGCCGACTTTGGCAGT 720
CDNANprvNep4    GACTGGGACGTTATGACCGACGCTGCCAAGCAGGCCCTCCAGAATGCCGACTTTGGCAGT 669

DNANprvNep4     GCCAACGTTCCCTTCAAGGACGGCAACTTTGAGACCAAGATTGAGGAGGCTTGGGTCTAA 780
CDNANprvNep4    GCCAACGTTCCCTTCAAGGACGGCAACTTTGAGACCAAGATTGAGGAGGCTTGGGTCTAA 729
```

E

```
DNANprvNep5     ATGCATTTCAACAACTTCATCGCCATGATCGCCGCCGCTTCCTGCGCTGTGGCTGCTCCT 60
CDNANprvNep5    ATGCATTTCAACAACTTCATCGCCATGATCGCCGCCGCTTCCTGCGCTGTGGCTGCTCCT 60

DNANprvNep5     GCTGCTGCCCCCGAGGCTGCCGCCGAGCAGATCGAGAAGCGCGCTGTTGTCGCTCACGAC 120
CDNANprvNep5    GCTGCTGCCCCCGAGGCTGCCGCCGAGCAGATCGAGAAGCGCGCTGTTGTCGCTCACGAC 120

DNANprvNep5     TCTCTCTGGCCCATGCCGGAGAGCGTCCGTGGCGGCACTGAAGGAAACGCCATTCGCCGC 180
CDNANprvNep5    TCTCTCTGGCCCATGCCGGAGAGCGTCCGTGGCGGCACTGAAGGAAACGCCATTCGCCGC 180

DNANprvNep5     TACGAGCCGTTCCTCCACATTGCTCACGGCTGCCAGTCGTACACCGCCGTCAACGCCCGC 240
CDNANprvNep5    TACGAGCCGTTCCTCCACATTGCTCACGGCTGCCAGTCGTACACCGCCGTCAACGCCCGC 240

DNANprvNep5     GGTGACACCAGGTGAGAACTCTCCTCCGCCCCCTTCGCAATTGCGCATCAACTGACATTC 300
CDNANprvNep5    GGTGACAC---------------------------------------------------- 248

DNANprvNep5     AACACAGCGGTGGTCTTCAGAACTCCGGCGGTGCCACTGCCGGCTGCCGTGATGACCGCA 360
CDNANprvNep5    ----CAGCGGTGGTCTTCAGAACTCCGGCGGTGCCACTGCCGGCTGCCGTGATGACCGCA 304

DNANprvNep5     GGGGCCAGACCTACGCCAGAGGTGCTTGGCACAATGGCCGCTACGCCATCATGTACTCCT 420
CDNANprvNep5    GGGGCCAGACCTACGCCAGAGGTGCTTGGCACAATGGCCGCTACGCCATCATGTACTCCT 364

DNANprvNep5     GGTACATGCCCAAGGACCAGATCTCCGACGGTGGTGCCAACGGTGGACACCGTCACGACT 480
CDNANprvNep5    GGTACATGCCCAAGGACCAGATCTCCGACGGTGGTGCCAACGGTGGACACCGTCACGACT 424

DNANprvNep5     GGGAGAACGTTGTTGTCTGGATTGATAACCCTCAGTCACCTCCTCTCCTCTACTTTAATA 540
CDNANprvNep5    GGGAGAACGTTGTTGTCTGGATTGATAACCC----------------------------- 455

DNANprvNep5     CCCCAAACTGGACAATAGCTGACTTCCCACACAGCGGCCAACGCGAACCCTCGTGTCTTC 600
CDNANprvNep5    -----------------------------------GGCCAACGCGAACCCTCGTGTCTTC 480

DNANprvNep5     GGTGCTGCTGCTTCTGGCCACGGCAGCTACAAGAAGACGACCAGCCCGCAGATGCGCGAC 660
CDNANprvNep5    GGTGCTGCTGCTTCTGGCCACGGCAGCTACAAGAAGACGACCAGCCCGCAGATGCGCGAC 540

DNANprvNep5     GGCAGCCGCCTCCAGGTCGAATACATGACCAACTTCCCCAGGAACCACGAGCTTCAGTTC 720
CDNANprvNep5    GGCAGCCGCCTCCAGGTCGAATACATGACCAACTTCCCCAGGAACCACGAGCTTCAGTTC 600

DNANprvNep5     AAGACCAGCCCTGGCCGCGACTTCTGGATGGTCGACTGGGCTACCCTGCCCGAGCCCGCC 780
CDNANprvNep5    AAGACCAGCCCTGGCCGCGACTTCTGGATGGTCGACTGGGCTACCCTGCCCGAGCCCGCC 660

DNANprvNep5     AGGAGGGCTCTCCAGGACACGTCTTTCGGCAGCGCGAACGTGCCCTTCAAGAACGGCAAC 840
CDNANprvNep5    AGGAGGGCTCTCCAGGACACGTCTTTCGGCAGCGCGAACGTGCCCTTCAAGAACGGCAAC 720

DNANprvNep5     TTCGAGAGCAACCTCGGCAAGGCCTGGATCTAA   873
CDNANprvNep5    TTCGAGAGCAACCTCGGCAAGGCCTGGATCTAA   753
```

Figure 1. Alignment of the DNA and cDNA sequence of *Neofusicoccum parvum* NLPs; **A**: NprvNep1, **B**: NprvNep2, **C**: NprvNep3, **D**: NprvNep4, and **E**: NprvNep5. Red boxes indicate the absence and presence of the introns in cDNA and DNA, respectively.

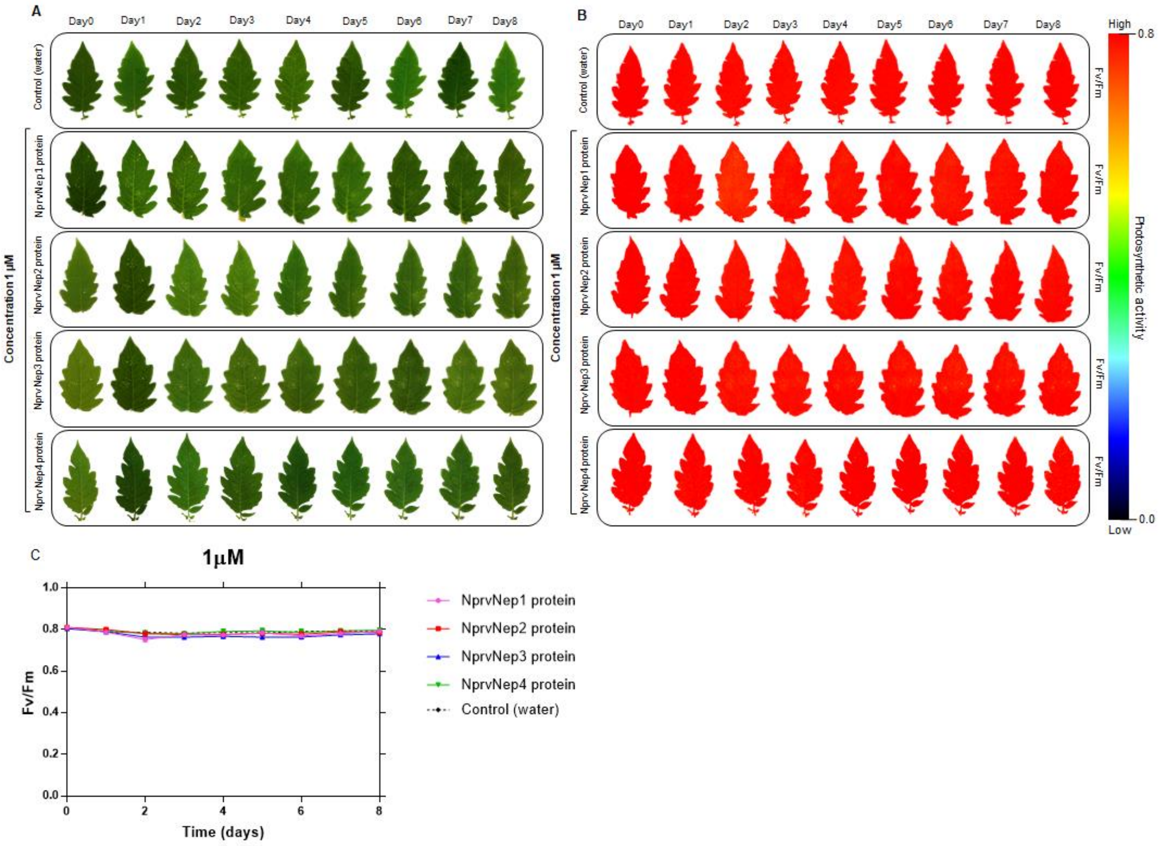

Figure 2. Effect of 1 µM recombinant NprvNeps on detached tomato leaves symptoms development. (**A**), chlorophyll fluorescence (**B**), and F_v/F_m (**C**). Ultra-pure water was used as a control. The color scale bar indicates the F_v/F_m intensity of the leaf pixels given in false colors from high (red) to low (black) values. All measurements were performed in biological triplicates and error bars show the standard deviation. Two-way ANOVA, followed by a Dunnett's multiple comparison test was used to determine the statistical significance of phytotoxicity of each protein within the same concentration against the control (**C**).

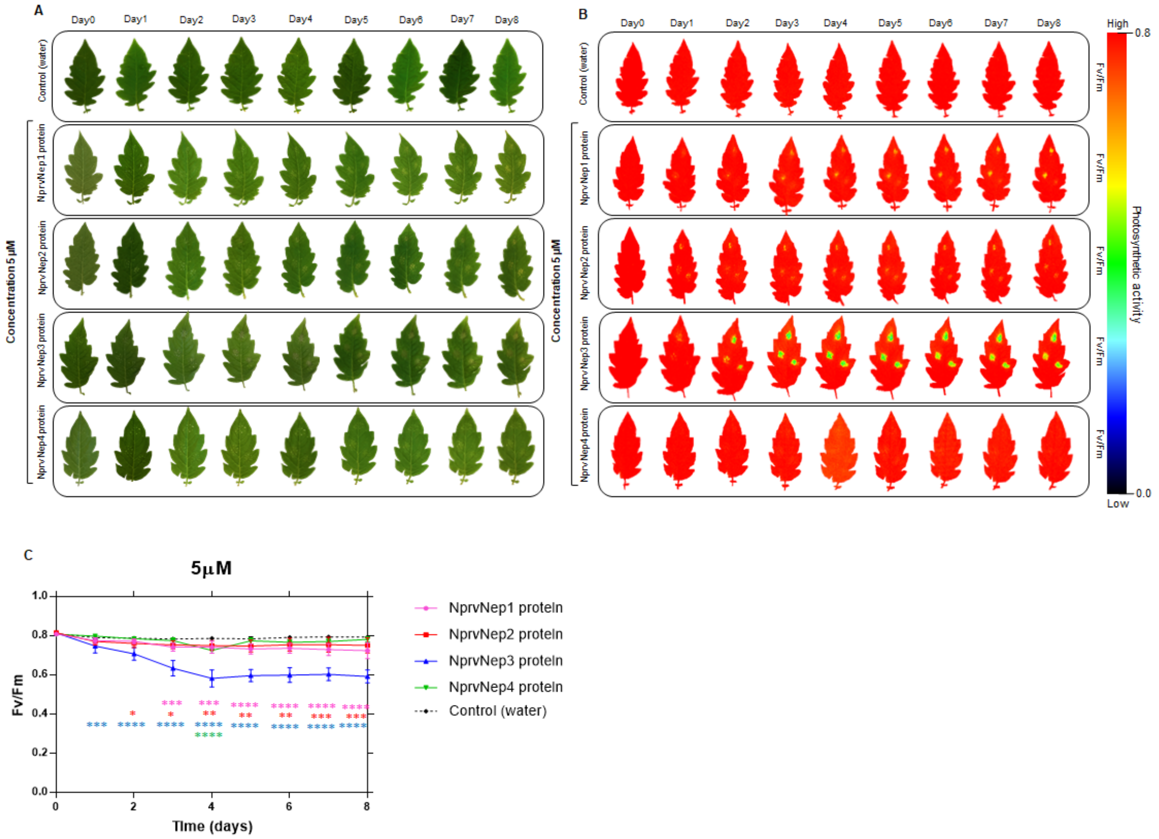

Figure S3. Effect of 5 μM recombinant NprvNeps on detached tomato leaves symptoms development. (**A**), chlorophyll fluorescence (**B**), and F_v/F_m (**C**). Ultra-pure water was used as a control. The color scale bar indicates the F_v/F_m intensity of the leaf pixels given in false colors from high (red) to low (black) values. All measurements were performed in biological triplicates and error bars show the standard deviation. Two-way ANOVA, followed by a Dunnett's multiple comparison test was used to determine the statistical significance of phytotoxicity of each protein within the same concentration against the control (**C**) (*$p<0.05$, **$p<0.01$, ***$p<0.001$, ****$p<0.0001$).

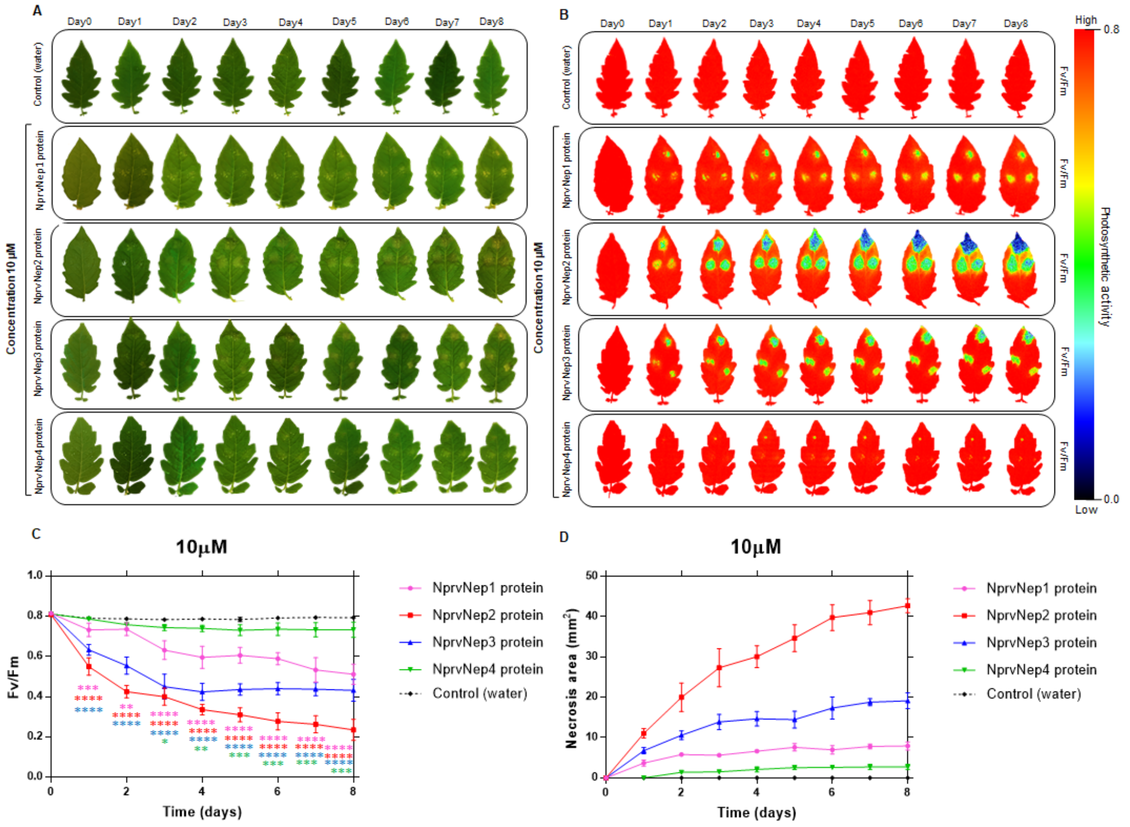

Figure S4. Effect of 10 µM recombinant NprvNeps on detached tomato leaves symptoms development (**A**), chlorophyll fluorescence (**B**), and F_v/F_m (**C**) and necrosis area (**D**). Ultra-pure water was used as a control. The color scale bar indicates the F_v/F_m intensity of the leaf pixels given in false colors from high (red) to low (black) values. All measurements were performed in biological triplicates and error bars show the standard deviation. Two-way ANOVA, followed by a Dunnett's multiple comparison test was used to determine the statistical significance of phytotoxicity of each protein within the same concentration against the control (**C**) (*p<0.05, **p<0.01, ***p<0.001, ****p<0.0001).

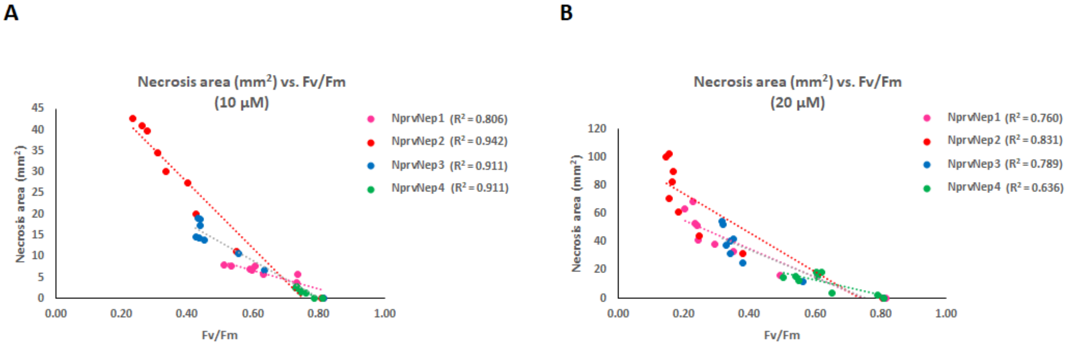

Figure S5. Scatter plot of necrosis area vs. Fv/Fm values for 8 days. The correlation between necrosis area and Fv/Fm values of detached tomato leaves treated with 10 µM (**A**) and 20 µM (**B**) recombinant NprvNeps (1-4) for 8 days is shown. Each point is the mean of biological triplicates.

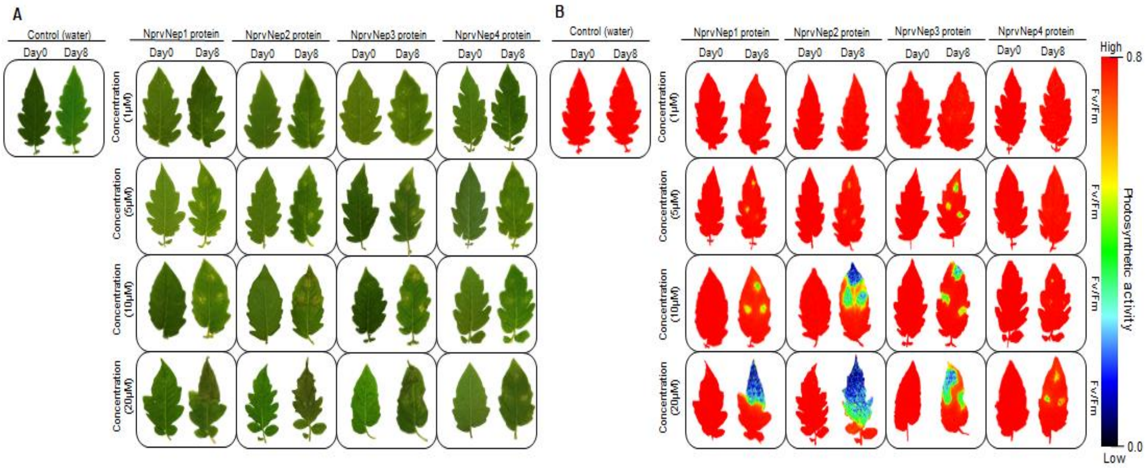

Figure S6. Toxicity of recombinant NprvNeps to detached tomato leaves evaluated by chlorophyll fluorescence. Effect of 1, 5, 10, and 20 μM recombinant NprvNeps on symptoms development (**A**), and chlorophyll fluorescence (**B**) at 0 and 8 dpi. Ultra-pure water was used as a control. The color scale bar indicates the F_v/F_m intensity of the leaf pixels given in false colors from high (red) to low (black) values. All experiments were performed in biological triplicates.

Table S1. Primers used for cloning and amplification.

Gene Name	Sequences (5′ to 3′)
NparvNep1	F: GCCCCGGTCGAGAAGCGC<br>R: CTACAACGCCGCTAACGCTAGGTTG
NparvNep2	F: GCGGTTGTTCAACGCCGCGG<br>R: TCACAACGACAGGCACAGGGG
NparvNep3	F: GCTGCCATCCAACGCCGC<br>R: TCAGACCTCAGCCTTGGCGAGG
NparvNep4	F: GCTCCCACGCAGAAGCTGAACG<br>R: TTAGACCCAAGCCTCCTCAATCTTGG
NparvNep5	F: GAGCAGATCGAGAAGCGCGCTG<br>R: TTAGATCCAGGCCTTGCCGAGGTTG

Table S2. The data of 6 *NprNep* genes.

Genes	GenBank No	Extracellular protein /signal peptide	Signal peptide length	Protein molecular weight (kDa)
NprvNep1*	gi∣615425645	Y	18	26.5
NprvNep2*	gi∣485922125	Y	21	27.6
NprvNep3*	gi∣485923842	Y	21	25.8
NprvNep4*	gi∣485928552	Y	18	26.2
NprvNep5*	gi∣485917230	Y	28	27.3
NprvNep6	gi∣615411409	N	0	17.9

A signal peptide of the NprvNep was predicted with the tool SignalP4.0. The SignalP Network predicted cleavage sites between 17 and 29 amino acid residues. * The *NprvNep* genes were selected for functional analysis. 'Y' has a signal peptide. 'N' has no a signal peptide.
